# Perceptions and experiences of engagement and participation in physical activity among older adults aging with long-term spinal cord injury

**DOI:** 10.3389/fspor.2026.1880498

**Published:** 2026-08-04

**Authors:** Kristina Fagher, Jan Lexell, Sophie Jörgensen

**Affiliations:** 1 Rehabilitation Medicine Research Group, Department of Health Sciences, Lund University, Lund, Sweden; 2 Department of Rehabilitation Medicine, Skåne University Hospital, Lund, Sweden

**Keywords:** adapted sport, ageing, physical activity, qualitative research, spinal cord injuries

## Abstract

**Background:**

Older adults aging with long-term spinal cord injury (SCI) are insufficiently physically active to reach health benefits. However, little is known about their perceptions of physical activity (PA) and barriers and facilitators to their PA engagement.

**Objectives:**

To explore perceptions and experiences of engagement and participation in PA among older adults aging with long-term SCI.

**Methods:**

We interviewed 74 men and women (56–88 years) from the Swedish Aging with Spinal Cord Injury Study (SASCIS) using a semi-structured interview guide. Interviews were transcribed verbatim and analysed with an inductive thematic analysis. Meaning units and codes were generated directly from the data without being influenced by pre-existing research frameworks. A latent approach was applied when forming themes, aiming to capture underlying assumptions and patterns.

**Results:**

Four different themes were revealed: “The multifaceted benefits of PA”, “PA comes in different forms; from incidental daily movement to formalized exercise”, “Restricted access: It is complex and difficult to be physically active” and “Unlocking potential: facilitators of PA”.

**Conclusions:**

This study highlights the essential role of PA in promoting well-being and independence for older adults aging with long-term SCI. Participants faced significant barriers, including accessibility, health-related challenges and lack of professional guidance, and emphasized facilitators such as the need for supervised training, social support and improved accessibility. Addressing these barriers and facilitators can create more inclusive opportunities for PA and exercise, ultimately supporting healthy, sustainable and active aging many years after SCI.

## Introduction

Aging with a spinal cord injury (SCI) becomes more common as health care develops and life expectancy increases (1). The combination of the aging process and the consequences of the injury increases the risk of developing premature metabolic and cardiovascular disease (2) as well as common SCI-specific secondary health conditions and physical dependence (1, 3), which together can negatively affect overall well-being. One of the most important strategies to counteract age-related changes after SCI is healthy lifestyle choices, such as regular physical activity (PA) (2, 4). However, persons with SCI face many barriers to engage and participate in PA (5) and can be considered among the most physically inactive group among people with lifelong disabilities (6–9).

One of the first longitudinal studies of PA participation in older adults aging with long-term SCI was pursued as part of the Swedish Aging with Spinal Cord Injury Study (SASCIS) (10, 11). Participation in leisure time PA (LTPA) was much lower than the current SCI-specific recommendations (12), with participants reporting a median amount of moderate-to-heavy LTPA of only 5 min/day (10). This overall level of participation was kept stable over six years, although there were substantial changes on an individual level (13). Moreover, about half of the participants performed no LTPA of moderate to heavy intensity at both time points (10, 13). Given the accelerated aging process after SCI, the benefits of PA might be even more pronounced in older adults aging with long-term injury. Hence, an obvious health concern is that the average older adult aging with long-term SCI is not reaching the amount or intensity of PA that is recommended to achieve health benefits (12).

Barriers to PA engagement and participation in people with SCI are fairly well researched. They include SCI-specific secondary health conditions and lack of motivation, accessibility, social support, knowledge and economical resources (5, 14). Still, there is a lack of studies specifically including older adults aging with long-term SCI. In the general, non-injured population, older adults are less likely to intend to participate in regular PA and prefer shorter and lower-intensity movement as compared to younger adults (15). Older adults can have negative emotions, such as fear or concerns about injury and/or falling and a grief of loss of previous activity, which can reduce motivation and limit engagement (16). Also motivators can differ between older and middle-aged adults. While younger adults can be more achievement-oriented, older adults may focus more on the social aspects of PA (17). Whether such mechanisms are present also in the older population aging with long-term SCI remains to be discovered.

To the best of our knowledge, no study has investigated perceptions and experiences together with barriers and facilitators to engagement and participation in PA among older adults aging with long-term SCI. Such knowledge would enable the design of appealing and realistic alternatives for PA in this group of people with SCI. The main aim of this qualitative study was to explore perceptions and experiences of engagement and participation in PA among older adults aging with long-term SCI.

## Methods

### Study design

This study is part of SASCIS, a longitudinal project aiming to understand factors associated with healthy aging in older adults aging with long-term SCI. The study design, method and quantitative results regarding PA have been described in previous studies (10, 11, 13, 18, 19). The quantitative data collection in SASCIS is extensive, with 12 different assessment tools administered at three time points (2011/2012, 2017/2018 and 2023/2024). In the second and third data collection, we added a few open-ended questions about perceptions and experiences of engagement and participation in PA, to include the participants’ own voices.

As the research area of PA among older adults aging with long-term SCI is understudied an exploratory thematic analysis according to Braun & Clarke 2006 was applied (20). This qualitative method is based on a theoretically flexible approach allowing researchers to identify, structure, analyse and describe data in-depth (20). As such, it was considered a suitable approach for our large sample and short interview guide.

Ethical approval was obtained from the Swedish Ethical Review Authority (No. 2016/911). All participants gave informed consent to participate in the study after receiving written and verbal information. The study follows the Consolidated criteria for reporting qualitative research (COREQ) (21), and the Helsinki Declaration for Medical Research Involving Humans.

### Data collection and participants

At baseline, the entire SASCIS project included 123 community-dwelling older adults with long-term SCI residing in southern Sweden. Participants were recruited through the clinical databases available at the SCI Unit at Skåne University Hospital, Sweden. The inclusion criteria were: i) age 50 years or older and ii) having a traumatic SCI or acquired nonprogressive SCI for at least ten years (11). In total, 74 participants (out of 78 eligible) consented to participate in an interview about PA in the second data collection. The characteristics of these participants are described in Table 1.

**Table 1:** Sociodemographics and injury characteristics of 74 older adults aging with long-term spinal cord injury.

Variable	Value
Sex, *n* (%)	
Male	51 (69)
Female	23 (31)
Age, y, mean ± SD; median, min-max	68 ± 8; 66, 56–88
Age at injury, y, mean ± SD; median, min-max	37 ± 15; 35, 7–71
Time since injury, y, mean ± SD; median, min-max	31 ± 11; 32, 15–55
Cause of injury, *n* (%)	
Traumatic^a^	47 (64)
Non-traumatic^b^	27 (36)
Level and severity of injury, *n* (%)^c^	
Tetraplegia AIS A-C	12 (16)
Paraplegia AIS A-C	24 (32)
All AIS D	38 (51)
Marital status	
Married/co-habiting/partner	46 (62)
Single	28 (38)
Vocational situation	
Working full-time/part-time	23 (31)
Disability pension/old age pension	51 (69)

SD, standard deviation

a Traffic/transportation (motor vehicle, train, bicycle), fall, workplace accident, diving accident, gunshot/assault/torture, other traumatic (e.g., sports, leisure activities).

b Spinal tumor, spinal disk herniation, spinal arteriovenous malformation, spinal infarction, spinal infection.

c AIS, American spinal injury association (ASIA) Impairment Scale describes the completeness of a spinal cord injury (SCI) and is based on the International Standards for Neurological Classification of Spinal Cord Injury (ISNCSCI).

As the qualitative questions were a part of a quantitative data collection the saturation principle was not used for this study. Data were collected by two researchers [SJ, physician specialised in SCI (last author) and UA, occupational therapist specialised in SCI] in the participants’ homes. Only the researchers and participants were present. A semi-structured interview guide (Appendix) focusing on the following themes were used; i) the importance of PA; ii) facilitators of PA and iii) barriers of PA. All interviews were transcribed verbatim. Data were analysed by SJ, KF (a physiotherapist and qualitative researcher working in adapted physical activity), JL (a senior physician, specialised in rehabilitation medicine and with extensive experience in qualitative research).

### Data analysis

**Figure 1:**
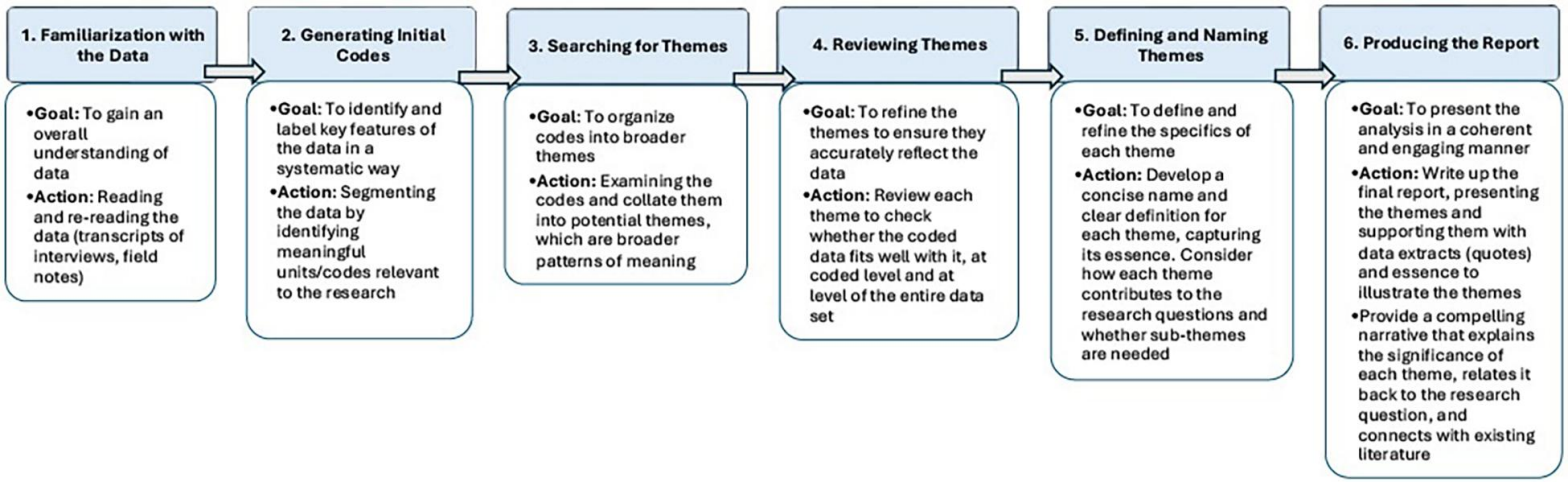
The six-step model described by Braun and Clark 2006 (20).

To analyse data, the six-step model described by Braun and Clark 2006 was followed (Figure 1). An inductive thematic analysis approach was applied to seek patterns. Meaning units and codes were generated directly from the data, without being influenced by pre-existing research frameworks. A latent analytical approach was used to develop themes that captured underlying assumptions and patterns across the participants’ accounts, and that the final themes were judged to provide a rich and comprehensive account of the phenomenon under study. To enhance the trustworthiness of the analysis, themes were continuously discussed and refined among the research team, including researchers with different background within the field, until consensus was reached. The themes were supported by representative quotations from participants, and an interpretive essence was developed to provide a deeper understanding of the meanings embedded within the data (20). Data and meaning units were extracted by KF, codes and themes were then formed by KF and SJ, and the final themes and draft of manuscript involved all three authors (KF, JL and SJ).

## Results

**Figure 2:**
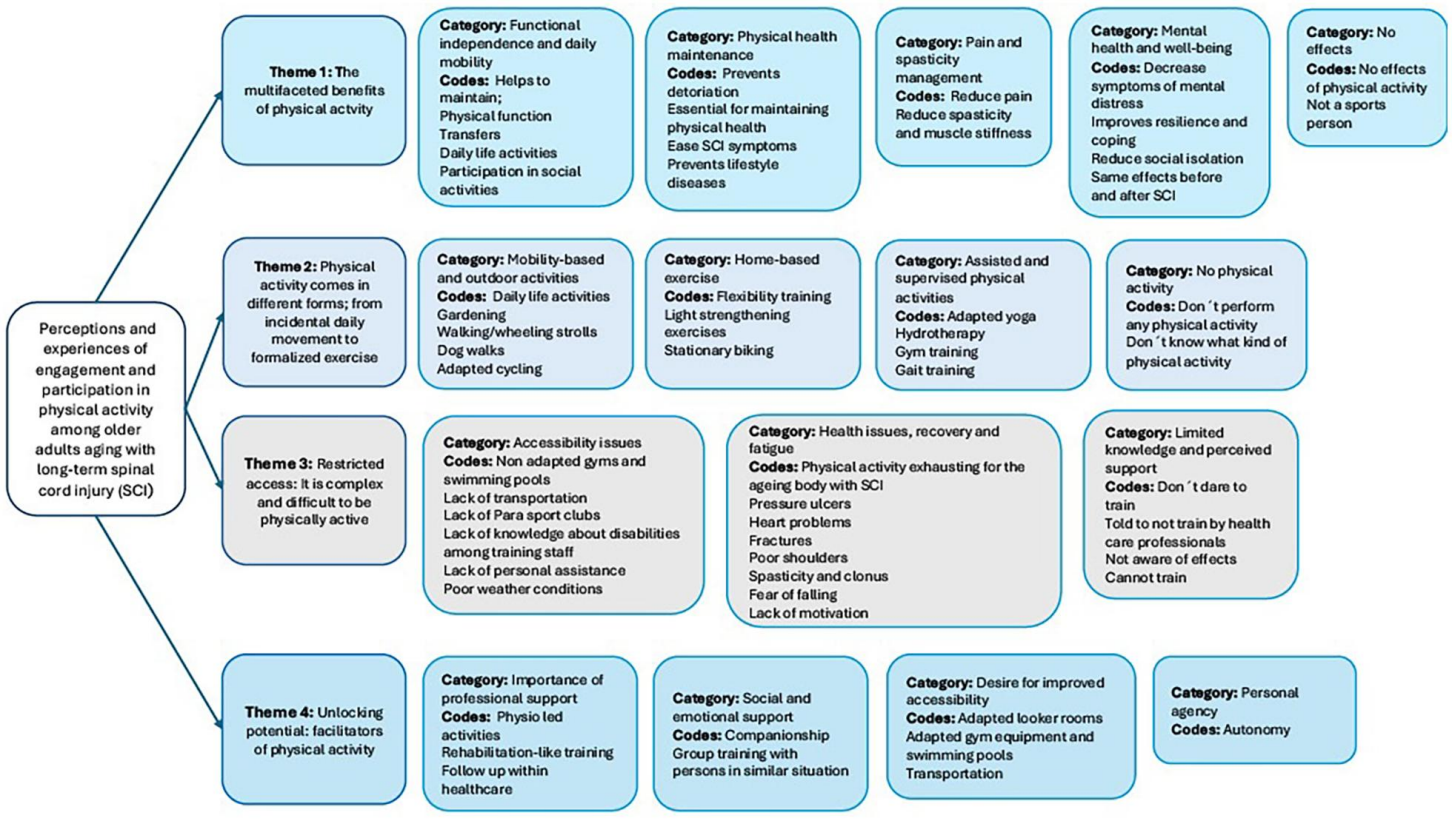
Perceptions and experiences of physical activity in older adults aging with long-term spinal cord injury: themes, associated categories and codes.

The thematic analysis revealed four different themes that are presented below together with key quotes. Associated categories and codes are presented in Figure 2.

### The multifaceted benefits of PA

Participants perceived that PA is essential for healthy aging among older adults aging with SCI. In particular, they expressed that PA is crucial for maintaining physical function and preventing deterioration associated with aging and SCI.

“PA is crucial for living a long life with SCI” (Participant 1)

“PA is so important—it's about being able to exist and not getting worse. Sitting in a wheelchair isn't natural; humans are meant to move. This applies to both the body and the brain” (Participant 50)

The perception was that one loses muscles so quickly when aging with SCI, and that it is even more important for persons with SCI to be physically active to maintain physical function, as compared to non-injured persons. The participants expressed that maintaining functional independence was a significant motivator for PA. It was described that PA allowed one to retain mobility skills necessary for transfers and daily life activities and reduce the need for external assistance. Staying physically active and strong was also important to be able to participate in the same social activities as other family members.

“Exercise helps me stay in control of my life and maintain independence in daily routines, like transfers” (Participant 2)

“For me, it’s very important to stay active because I want to have a mobile life and be able to join my family and friends in activities they do” (Participant 41)

Furthermore, it was described that regular PA helped to preserve cardiovascular health, lung function and bowel function. Several participants emphasized that PA helps them control chronic pain and other common secondary health conditions such as muscle stiffness and spasticity. Some participants mentioned specifically that more structured exercise assisted them to regulate weight, manage diabetes, prevent infections and improve bone density.

“Now that I have been exercising over the past year, I have felt more energetic, had less spasticity, and fewer infections (both febrile urinary tract infections and colds), and my digestion is working better” (Participant 3)

“If I don’t exercise, I get more pain in my shoulders and back” (Participant 28)

Lastly, it was perceived that PA serves as an emotional outlet. Participants described that staying active improves overall mental well-being, and enables one to manage stress, anxiety, depression, emotional resilience, and sleep as well as to cope with the situation. Their experience was that group PA, for example at a rehabilitation clinic, led to a feeling of social inclusion and prevented social isolation. Also, outdoor activities, such as being out with the dog, was perceived as leading to social engagement. One participant specifically expressed that these positive effects of PA were the same as prior to acquiring the SCI, which contributed to a sense of normality. Others described PA as a previously valued habit, and they felt great sadness when they could no longer participate as they would like to.

“Exercising reduce my mental distress and it helps me cope with my situation—aging with a disability” (Participant 4)

Some participants described that they had tried to participate in PA, but that it resulted in no positive effects, and therefore they had stopped. One participant had the perception that exercise is harmful when aging with SCI.

“I've been doing it (exercised) for a long time. But. I haven't gotten better from it, I haven't. No, I haven't. So that's the reason why I've stopped. I would have gladly done it if I had experienced improvements” (Participant 64)

“I don't want to do any exercise because it could harm me” (Participant 2)

Taken together, this theme emphasizes the mostly holistic impact of PA on both physical and mental well-being as well as independence and autonomy among older adults aging with long-term SCI.

### Physical activity comes in different forms; from incidental daily movement to formalized exercise

Regarding the types of PA, most participants described that they were engaged in low intensity mobility-centred activities, such as walking with aids (e.g., walkers, rollators) or wheeling. Sometimes these activities were described as planned PA sessions, but most often these activities were related to daily life activities such as grocery shopping, gardening or cleaning. For example, a participant described that walking 600 steps a day during daily life activities was the PA for the day. Furthermore, many participants perceived outdoor activities such as walking and wheeling with their dog or gardening as enjoyable.

“I am grateful for having my dog; if it weren’t for that, I wouldn’t even go outside” (Participant 115)

Another common PA modality was home-based exercise, including stretching, strength training with resistance bands, and using other home equipment (e.g., stationary bikes or hand cycles). Participants perceived that this type of PA can be easily adapted to personal comfort levels while reducing the risk of injury. However, several participants also struggled with motivation to perform such activities.

“I do some flexibility training in my bed, but it is difficult with the motivation, and I don't know anyone in my situation who goes to the gym, participates in organized sports or does online training” (Participant 15)

Some participants participated in structured sessions with physiotherapists within the health care system, such as strength training, gait training, hydrotherapy, adapted yoga and standing exercises. This type of supervised exercise was perceived as enjoyable, safe and effective. However, several participants felt they needed more supervised exercise than they received, and the perception was that the possibilities for supervised exercised within the healthcare system decreased every year.

“Walking on the treadmill at the primary health care centre strengthens me a lot, but then I need help from the physiotherapist, and unfortunately there have been restrictions on how much you can exercise within health care, they don't have time for us anymore. I used to go every week, now only once a month” (Participant 32)

A few participants trained by themselves at a regular gym. None of the participants participated in organized Para sport, but some had a wish to.

“The only thing I can do is to pull on some resistance bands at home, I wish I could be a little more physically active in a varied way, such as skiing. But it is what it is” (Participant 59)

In summary, this theme captures the range of physical activities that participants engage in, from incidental daily movement to more structured exercise and therapy routines.

### Restricted access: It is complex and difficult to be physically active

Participants described that they wanted to be physically active, but there were too many physical barriers for older adults aging with long-term SCI. Examples of a major barrier was physical accessibility at gyms, swimming pools and other training facilities. Problems included lack of adapted gym equipment and swimming pools, inaccessible changing rooms, and stairs that did not allow wheelchair access. One participant expressed that persons with an impairment are not even welcome to some training facilities, and another one described that staff working at many training facilities do not have knowledge about persons with a disability. Other barriers were lack of or long transportations, no personal support during training, financial costs, no Para sport clubs in the hometown and bad weather.

“I would like to exercise, but the big problem is getting to the training. I need both adapted transportation and personal assistance” (Participant 21)

“I want to participate in Para team sports, but there is no adapted training where I live, I have to drive 400 km to participate in a Para team sport” (Participant 73)

Another common experience was that health issues, prolonged recovery time and fatigue were barriers to participation in PA. It was perceived that PA for a person aging with SCI can be exhausting, and that it could take days to recover from a PA session, especially if it also included long transportation, inaccessible facilities and personal assistance.

“I used to swim, but I can't manage it anymore because it's so difficult/tiring to go to the pool and get dressed etc.. older people with SCI need so much more support when exercising, otherwise it will just lead to fatigue” (Participant 113)

Several participants expressed that they were waiting for health improvements, such as healing of pressure sores, fractures or shoulder problems, before they could engage in PA. Some participants described that health issues related to spasticity or heart disease restricted them from participation in PA, and some did not dare to be active because of their health issues.

“I do actually train, but over the past year I haven't been able to do it because of my pressure sores” (Participant 5)

A perception among the participants was that the aging body was giving up and that one must fight hard for everything. Examples were factors related to loss of function in the upper extremities, secondary health conditions and fear of falling. Participants also spoke about a sense of loss and sorrow of not being able to do certain activities anymore, such as cycling or playing golf, and therefore they choose not to participate in any activities.

“I fight every day to not become an old, lazy person. It's an ambition and a struggle, I fight. But what used to be easier when I was a bit younger, today it's much, much harder. But somehow, you have to put in an effort to be active.. I mean, in terms of willpower, you have to push much, much harder when aging with SCI” (Participant 76)

“I don't dare to train because of my health problems, before I used to do wheelchair dancing, it was really fun, but now I don't dare” (Participant 27)

“All the time I think I would fall, and I am so afraid of falling, it really limits me from being physically active” (Participant 2)

Participants also expressed a sense of resignation, describing that there is “no need” to perform PA when aging with SCI. One participant described that health care professionals had told them not to engage in PA due to weak shoulders. Furthermore, it was expressed that physicians do not take evaluations and recommendations about PA seriously.

“It's not worth striving for starting to exercise.. trying to keep striving because I can't achieve it anyway. Then it's not worth thinking about it because it just.. makes you feel sad” (Participant 52)

“The physiotherapist has told me to exercise, but the doctors are completely uninterested. They probably think, like most people, that when you're a bit overweight and in wheelchair, it's your own fault. I've asked for help from my doctor over the past three four years. One has to fight for help, but I can't manage that anymore” (Participant 90)

Several participants expressed that they did not know about the benefits of PA and what kind of PA they could take part in. Moreover, participants mentioned lack of motivation, self-discipline and time, often linked to not seeing the benefits or the enjoyable aspects of PA.

“It would really help me to find motivation for PA if I also knew about the effects” (Participant 83)

“I don't know what type of PA I could do, I would need medical and professional advice” (Participant 62)

In summary, the responses reflect a mix of barriers, both physical, environmental and psychological, that restrict older adults aging with long-term SCI from participating in PA.

### Unlocking potential: facilitators of physical activity

To facilitate PA among older adults aging with long-term SCI, participants described that professional support is crucial. It was expressed that one needs training supervised by physiotherapists, as they have the expertise in guiding persons with SCI. Furthermore, it was perceived that the “rehabilitation like training” they received during the initial rehabilitation phase was more suitable than exercising at a sports club or gym.

“Training with a physiotherapist would help, it would be regular and a bit forced. It feels good with the equipment they have, and there is also someone who monitors and motivates you” (Participant 62)

Further supporting the need for supervision and guidance, participants expressed that they wanted to be more physically active, but that they did not know how. Several participants spoke about the importance of health care professionals regularly assessing, evaluating and recommending PA. A perception was that communication with professionals about one's PA level could improve motivation and personal agency. Some participants also expressed that more structured training in one's home could facilitate PA and help avoiding fatigue.

“There isn't much information or recommendations about what one can and should do, based on the conditions one has” (Participant 51)

These experiences were accompanied by the importance of having social and emotional support. Participants spoke about having someone, e.g., a personal assistant or family member, that could accompany them during PA would increase their motivation to exercise. A desire was to be part of a training group with peers in a similar situation.

“When I was young, I played team sports, but I lost that opportunity when I was injured. I wish I could exercise in a group with individuals in the same situation as mine again. When you're part of a group, it's easier to find motivation, but when you're alone, it's hard to take that initiative” (Participant 49)

Another important aspect was improved accessibility. Better access to warm pools, better transportation possibilities to training facilities, accessible locker rooms and specialized gym equipment for wheelchair users were emphasised.

“Sometimes I would like to go to the gym, but I can't really get into the gym here with my wheelchair due to lack of accessibility” (Participant 21)

There were also a few participants who perceived that there is nothing that can facilitate participating in PA as they are not interested in taking part.

“There is nothing that would make it easier for me to participate in training or PA, and I don't feel a need for it either” (Participant 66)

Taken together, this theme describes that to facilitate PA among older adults aging with long-term SCI there is a need for both external support (professional, social, and environmental) and internal drive (motivation and personal agency).

## Discussion

This study explored perceptions and experiences of engagement and participation in PA among older adults aging with long-term SCI. The results indicate that there are several barriers related to restricted access, few opportunities and lack of knowledge about PA. At the same time, participants expressed that they had a wish that they could be more physically active, and that they were aware that PA lead to several physical and mental health benefits.

### PA for healthy aging with long-term SCI

The participants described that PA led to several positive effects such as improved physical and mental health, improved function, less pain and spasticity as well as a reduction of physical deterioration. We have previously reported in the SASCIS population an association between more LTPA and lower BMI and waist circumference (19) as well as fewer depressive symptoms (18). Thus, PA can be considered an important factor to preserve health and function in the aging population with long-term SCI. It is also well established that PA is promoted as a tool to prevent all-cause mortality in persons without a disability (22). Even though persons with a disability are understudied compared to the general population, meta-analyses have shown that PA has beneficial effects on musculoskeletal fitness, cardiovascular fitness, cardiometabolic risk factors, and brain and mental health outcomes in this population (23). Still, many people with disabilities do not meet PA guidelines despite being at higher risk of serious health problems related to inactivity compared to people without disabilities (23). One way to promote PA is to inform about the amount and intensity required to reach beneficial health effects (24). Yet, there are few studies of the effects of PA and exercise among older adults aging with long-term SCI. Indeed, there was a lack of representation of older adults in the evidence base for creating the SCI-specific PA guidelines (4), and adaptations might be needed to suit the specific physiological changes occurring when aging with long-term SCI.

The results of the present study imply that older adults aging with long-term SCI may have an unmet need for more individualized and supervised PA. Given the increased risk of developing premature lifestyle-related disease in this population (2), tailoring PA counselling and promotion strategies to their needs and preferences should be prioritized. It is important to be aware that health benefits probably can be achieved with less than the amount stipulated in guidelines and that some PA is better than none (23).

### Health care system responsibilities to facilitate PA

Participants expressed that there is a need for health care professionals to better follow up on and recommend PA. A perception was that health care professionals sometimes are not interested in or do not take PA seriously. Cunningham and O'Sullivan (25) reported that the awareness of guidelines regarding PA for the general aging population vary among health care professionals, and that assessment of and support for PA is not a routine during regular follow-up visits. It was suggested that healthcare professionals should take a greater responsibility in promoting PA, especially for older adults (25). However, a challenge is that health care professionals report limited time and possibilities to be able to include PA in their assessments (26). Given the financial priorities and lack of resources in modern health care, there is a need for a closer collaboration between health care, society and private enterprises to develop alternative ways by which people with life-long disabilities can be stimulated to engage in PA. People with a disability should be able to use accessible facilities where staff is knowledgeable in promoting and following-up PA.

Nevertheless, as PA is a non-invasive, non-pharmacological and cost-effective intervention for managing chronic disease (27), we strongly believe that PA evaluation and promotion should be more prioritised within health care and rehabilitation, and be an integrated part in the long-term management after SCI. As the proportion of persons living with a long-term SCI into old age increases, preservation of health and function in this population may have implications both for the individual, the health care system and for reducing societal costs. It has been suggested that interventions preventing age-related conditions after SCI should be implemented at an early stage to allow “successful ageing” in advance (1), preferable already in middle-age. Thus, there is a call for stakeholders to take a larger responsibility in education, funding and evaluation of interventions to promote PA in this population. As participants in this study also described that they did not know what kind of PA they could or should do, there is a further call for the research community to develop, implement and evaluate evidence-based interventions targeting older adults aging with long-term SCI.

### Barriers and facilitators to PA participation

An important aspect to consider is behaviour change related to participation in PA since participants expressed that there were several barriers related to themselves. It has been proposed that changing health behaviour requires action at three levels: individual, social/environmental and institutional (27). To change individual behaviour, one requires knowledge and skills (27). In the present study, several participants said that they wanted to be physically active but did not know what to do, whereas some participants expressed that PA is not necessary for persons aging with SCI. Hence, it can be recommended that stakeholders (such as Public Health Authorities, rehabilitation departments and health care professionals) proactively work towards increasing health literacy in the aging population with SCI. Even in high-resourced settings such as Western and Northern Europe, many persons have limited health literacy skills (28). This is also reflected in our findings as some participants were not aware of the positive health effects of PA.

In the present study, the participants described that social factors, such as performing PA together with family members or as dog walks, were important facilitators to participation. In a younger population with SCI it has been shown that peers are important as knowledge translators and motivators to participation in PA (29). Hence, it could be assumed that peer support, involving another older adult with long-term SCI who share experiences and skills could facilitate engagement in PA. Peer support programs helping people with heart disease to maintain PA after cardiac rehabilitation have been successful, especially for older persons (30).

Participants in this study mentioned several barriers related to the physical environment, such as non-adapted training facilities and lack of transportation. In Sweden, there is a law stating that all built environments should be accessible to persons with various disabilities (31), and the results from this study indicate that this is only partly met. Sweden has recently been criticised by the UN Convention on the Rights of Persons with Disabilities (CRPD), stating that there is a need to better fulfil rights and opportunities for persons with disabilities (32). Hence, there is a need for stakeholders at an institutional level, both within health care and sports, to further assure that training facilities are accessible and that persons with a disability, if needed, have access to transportation and personal assistance.

Some participants did not want to participate in PA, and it is important to remember that older adults aging with long-term SCI have their own experiences, motivators and thoughts of healthy aging and longevity. As researchers in PA, we often tend to develop guidelines and expect individuals to follow them. However, it is also necessary to embrace an enactive view of the human development and cognition during aging (33), and especially among persons living with a life-long disability. Hoekstra and colleagues presented a useful example on how to address preferences, concerns and feedback from the general population with SCI when translating scientific exercise guidelines into guidelines that can be used in the community and clinical practice (34).

### Methodological considerations

The interviews were part of a large quantitative data collection within the SASCIS project, and together with this data collection a short qualitative part was included to broaden the understanding of PA. The interview guide was short (consisting of the three themes (importance of physical activity, facilitators, and barriers) but given the large number of participants and follow-up prompts, we believe that the data provided a comprehensive understanding of perceptions and experiences of engagement and participation in PA in this previously unresearched population. Some of the participants’ perceptions may be a Swedish phenomenon, and it could be recommended that similar studies are conducted in other cultural contexts to enable transferability. Most participants were informative and open to share their perceptions. Hence, the credibility is considered good. However, a concern in qualitative research is the researchers’ understanding of what the participants are trying to communicate, and we believe it is a strength of the SACSIS project to have included both qualitative and quantitative data collection methods to improve the understanding of PA in this population. To assure dependability, the two interviewers were health care professionals familiar with persons living with SCI, and all authors participated in the analysis. However, a limitation is that two interviewers were involved in data collection, which could have caused variability in the way questions were asked. To minimize researcher bias in the data analysis, regular peer discussion and triangulation of findings were held within the author team. The overall trustworthiness is considered good, but studies in other settings with more detailed interview guides are preferable.

## Conclusion

This study highlights the essential role of PA in promoting well-being and independence for older adults aging with long-term SCI. Participants recognized its benefits for physical function, mental health, and daily life activities but faced significant barriers, including accessibility, health-related challenges, and lack of professional guidance. To overcome these barriers, participants emphasized the need for facilitators such as supervised training, social support, and improved accessibility. They also highlighted the importance of healthcare professionals in recommending and encouraging PA. Addressing these barriers and facilitators can create more inclusive opportunities for PA and exercise, ultimately supporting healthy, sustainable and active aging many years after SCI.

## Data Availability

The raw data supporting the conclusions of this article will be made available from the corresponding author upon reasonable request.
