# Supplementary material for: Perceptions and experiences of engagement and participation in physical activity among older adults aging with long-term spinal cord injury
Source: Front Sports Act Living. 2026 Aug 4;8:1880498. doi: 10.3389/fspor.2026.1880498 (PMC13481554; doi:10.3389/fspor.2026.1880498)
Supplement: Supplementary file 1 [file Supplementaryfile1.docx]

**Appendix**

Semistructured interview guide

"How important is it to you to be physically active or exercise? - Please rate on a scale from one to ten, where 1 means not important at all and 10 means very important."

"Is there anything that limits your participation in physical activity?"

"What would support or increase your participation in exercise and physical activity?”

Probes: “Could you please tell me more about that?” “When you say that…. Could you develop that further?” “You talk about the…, how does that affect your ability to engage in PA?”
